# CT Urography-Based Radiomics to Predict ISUP Grading of Clear Cell Renal Cell Carcinoma

**DOI:** 10.7150/jca.105173

**Published:** 2025-01-06

**Authors:** Panpan Jiao, Bin Wang, Xinmiao Ni, Yi Lu, Rui Yang, Yunxun Liu, Jingsong Wang, Haonan Mei, Xiuheng Liu, Xiaodong Weng, Qingyuan Zheng, Zhiyuan Chen

**Affiliations:** 1 Department of Urology, Renmin Hospital of Wuhan University, Wuhan, Hubei, 430060, China.; 2 Department of Urology, Suizhou Central Hospital, Suizhou, Hubei, 441300, China.; 3 Institute of Urologic Disease, Renmin Hospital of Wuhan University, Wuhan, Hubei, 430060, China.; 4 Department of Ophthalmology, Renmin Hospital of Wuhan University, Wuhan, China.

**Keywords:** Clear cell renal cell carcinoma, ISUP grading, Radiomics, Machine learning, Artificial intelligence

## Abstract

**Purpose:** Exploring the value of predicting the WHO/ISUP grade of clear cell renal cell carcinoma (ccRCC) using computed tomography urography (CTU) images, providing valuable recommendations for the treatment of ccRCC.

**Method:** CTU images from the Renmin Hospital of Wuhan University (RHWU) cohort, including 328 patients with ccRCC, were retrospectively collected. The corticomedullary (CMP) phase features of ccRCC were extracted from the CTU images using the Pyradiomics package, and key features were selected through the Least Absolute Shrinkage and Selection Operator (LASSO) regression. The 328 patients were split into training and testing sets in a 7:3 ratio. 175 patients from the The Cancer Genome Atlas (TCGA) cohort were used for the external validation set. Various models, including Logistic Regression (LR), Multilayer Perceptron (MLP), Support Vector Machine (SVM), and eXtreme Gradient Boosting (XGBoost), were employed to predict the ISUP grade. SHAP analysis was then used to visualize the performance of the best model.

**Results:** A total of 1,218 features were extracted using the Pyradiomics package, with 20 features selected for model training through LASSO analysis. In the training set, the AUC for the LR model was 0.88 (95% confidence interval [CI] 0.84-0.91), for MLP it was 0.89 (95% CI 0.86-0.93), for SVM it was 0.86 (95% CI 0.83-0.90), and for XGBoost it was 0.96 (95% CI 0.92-0.99). In the testing set, the AUC for LR was 0.79 (95% CI 0.73-0.85), for MLP it was 0.78 (95% CI 0.72-0.83), for SVM it was 0.78 (95% CI 0.73-0.82), and for XGBoost it was 0.80 (95% CI 0.75-0.85). In the validation set, the AUC for LR was 0.74 (95% CI 0.68-0.79), for MLP it was 0.68 (95% CI 0.63-0.73), for SVM it was 0.67 (95% CI 0.64-0.71), and for XGBoost it was 0.78 (95% CI 0.74-0.83). XGBoost demonstrated superior performance, with a sensitivity of 0.99 (95% CI 0.96-1.00) in the training set, 0.92 (95% CI 0.88-0.97) in the testing set and 0.91 (95% CI 0.86,0.95) in validation set. SHAP analysis revealed that the wavelet-LHL_glcm_Idn and wavelet-LHL_glrlm_LongRunEmphasis features played pivotal roles in the classification task.

**Conclusion:** In this study, we employ an artificial intelligence model to conduct non-invasive ISUP grade prediction on preoperative CTU images of ccRCC, thereby aiding clinical decision-making. Additionally, we uncover that the radiomics features extracted from the CMP phase of CTU images hold promise as potential biomarkers for grading ccRCC.

## Introduction

Renal cell carcinoma is a common tumor originating from renal tubular epithelial cells^1^. In 2020, approximately 431,000 new cases of renal cancer were reported globally, with 271,000 occurring in men^2^. Studies indicate that the global incidence of renal cancer continues to rise^3^. Clear cell renal cell carcinoma (ccRCC) is the most prevalent type, accounting for around 70% of all renal cancers^4^. The diagnosis of ccRCC primarily relies on traditional histopathological examination. Evidence suggests that morphological changes in the nucleolus can be observed in cancerous tissues, indicating a diagnosis, and the relationship between nucleolar hypertrophy and poor prognosis has drawn increasing attention from pathologists^5^. Numerous studies have identified nucleolar hypertrophy as a prognostic indicator of poor outcomes in cancer patients^6^. Additionally, nuclear morphological characteristics can help distinguish low-grade from high-grade tumors, with nuclear atypia often linked to malignancy^7^.

The grading system was introduced by the International Society of Urological Pathology (ISUP) at the Vancouver meeting in 2012 and is recommended for use by the World Health Organization (WHO). The degree of nucleolar prominence is assessed to determine grades 1 to 3, while the presence of highly atypical pleomorphic cells and/or sarcomatoid or rhabdomyomatous morphology indicates grade 4^8^. In comparison to the Fuhrman system, the ISUP grading system may place greater emphasis on evaluating tumor necrosis and sarcomatoid and rhabdomyomatous features, which are not adequately represented in the Fuhrman system^9^. The ISUP grading system is easier to apply and demonstrates greater reproducibility and clinical relevance^10^. Percutaneous pathological biopsy is a common method for preoperative grading of ccRCC but often results in bleeding. Furthermore, due to the heterogeneity of ccRCC, inconsistencies can occur between biopsy and resection samples within the ISUP grading system. Thus, there is an urgent need to develop a new, non-invasive, and effective method for determining the histological grade of ccRCC.

In recent years, numerous studies have explored the intersection of artificial intelligence technology and ccRCC. Yeh, F.C. *et al.* achieved segmentation and classification of nuclei in pathological images^11^. Pan, L. *et al.* utilized multimodal MRI radiomics to predict the Fuhrman grading of ccRCC^12^. Zhang, Y. *et al.* developed a multi-information fusion model that integrates tumor CT features and biochemical indicators to predict the Fuhrman grading of ccRCC^13^.

However, there have been no large-scale cohort studies investigating the ISUP grading of ccRCC. In this study, we aimed to utilize radiomics and machine learning techniques to predict the ISUP grading of ccRCC, providing a non-invasive method for determining the ISUP grade. This approach might facilitate improved clinical management and enhance patient prognosis.

## Methods

**Figure 1:**
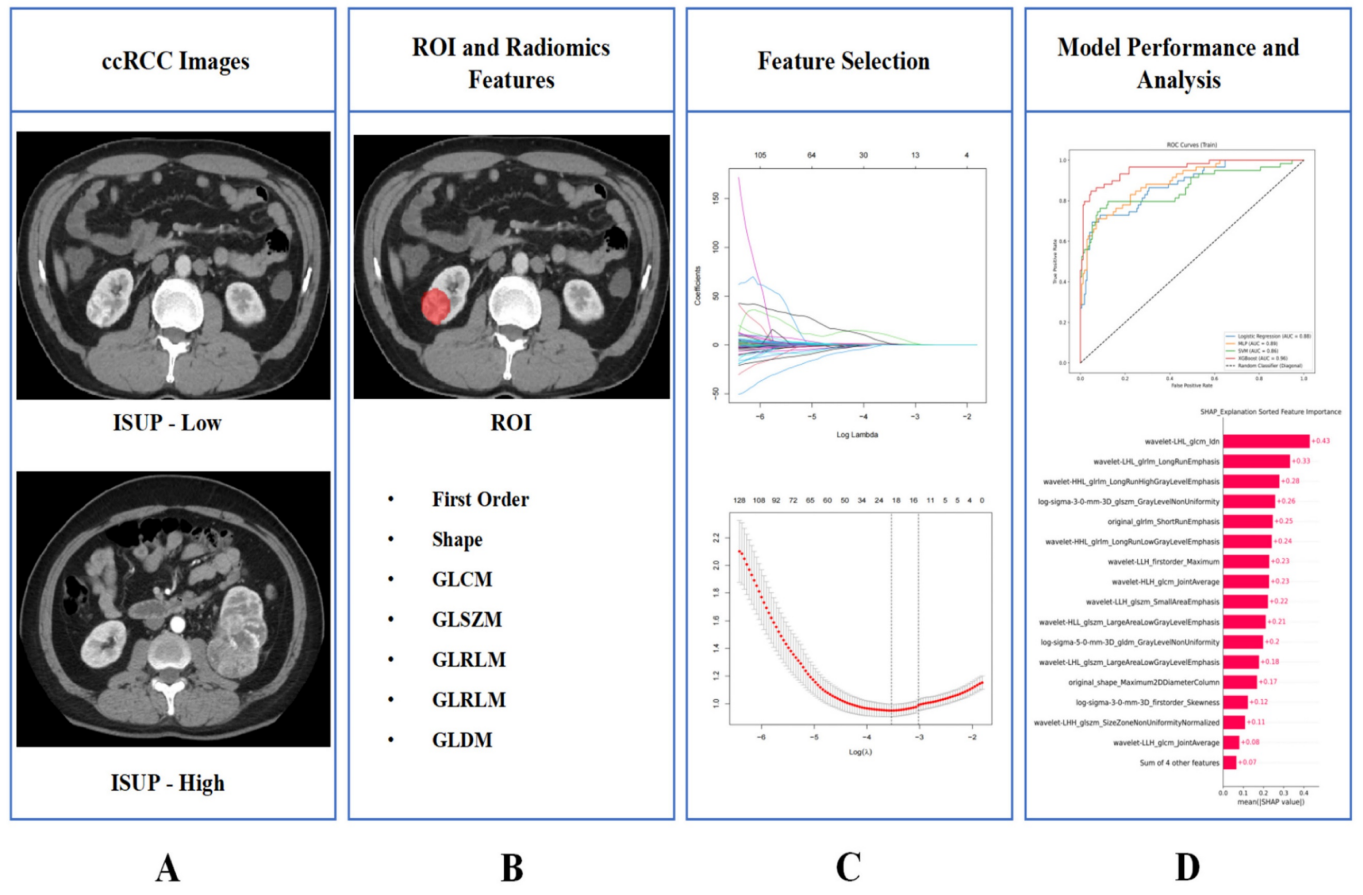
The entire workflow of the study. **(A)** Collect images of ccRCC; **(B)** ROI was determined and image omics feature extraction was performed; **(C)** Radiomics features screening; **(D)** Train the model and demonstrate its interpretability.

The design process of the entire study is illustrated in **Figure 1**.

### Study population

**Figure 2:**
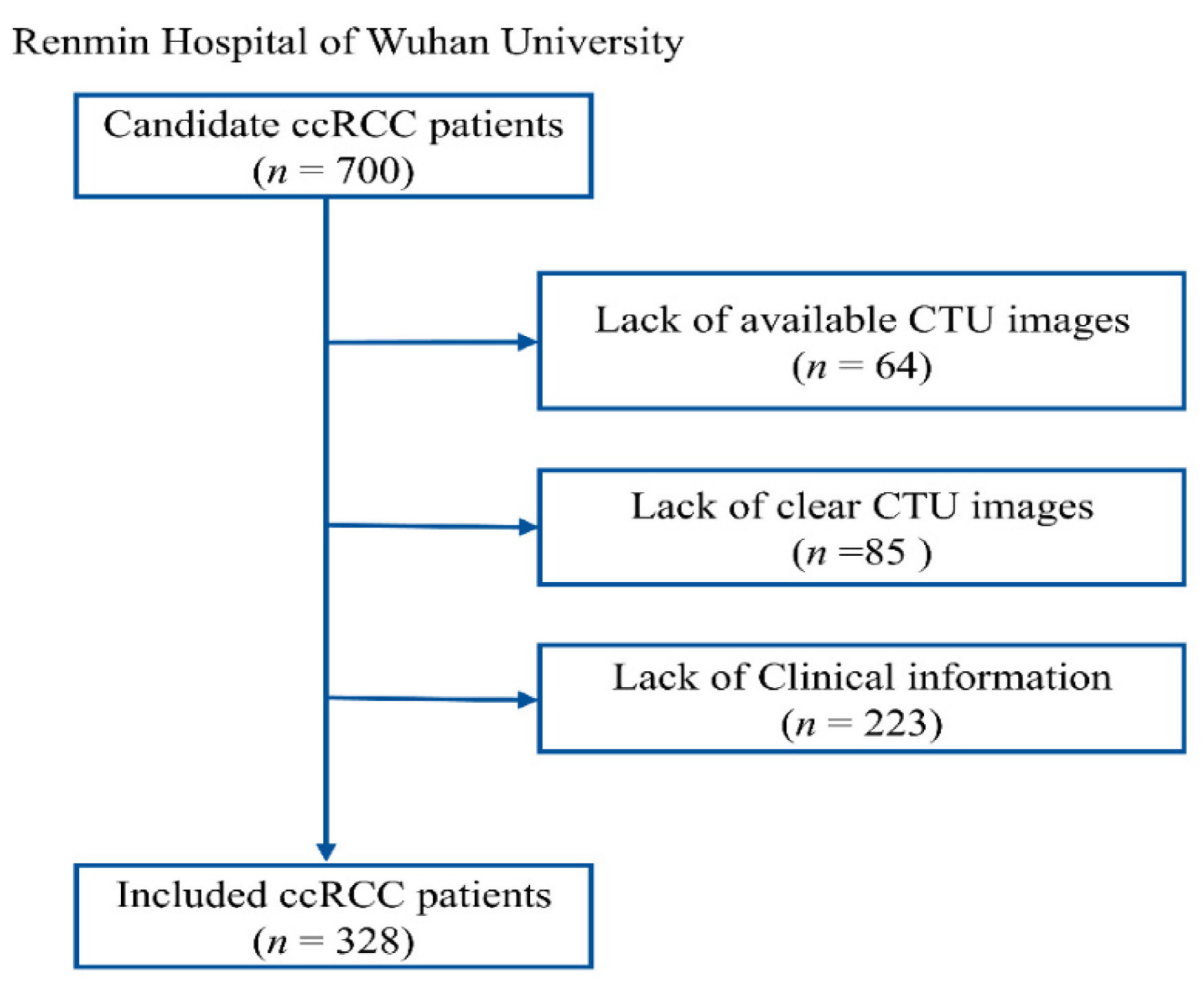
Recruitment process of RHWU cohort.

This study adhered to the Helsinki Declaration and received approval from the Clinical Research Ethics Committee of Renmin Hospital of Wuhan University (RHWU), with approval number WDRY2022-K077. We recruited 700 patients who underwent surgery for ccRCC at RHWU between 2020 and 2024, all of whom were confirmed to have ccRCC through postoperative pathological diagnosis. Among these, 85 cases had poor image quality, 64 cases had missing CTU images, and 223 cases had incomplete clinical information, leading to their exclusion from the study. 175 patients from the The Cancer Genome Atlas (TCGA) cohort were used for the external validation set. Only patients with a confirmed pathological diagnosis of ccRCC, known ISUP grading status, no prior targeted therapy or chemotherapy, no other tumors, and complete data on age, gender, and TNM staging were included. The entire patient recruitment process is illustrated in **Figure 2.**

### Image analysis

Thin-slice CTU images with a slice thickness of 0.625 mm were collected from patients with a confirmed diagnosis of ccRCC. Two radiologists, each with seven years of experience, were recruited to independently and systematically analyze the CTU images, without access to the clinical information. In cases where discrepancies or doubts arose, a third radiologist with 12 years of experience would review the images and make the final determination. All CTU images were then saved in DICOM format.

### ISUP assessment

We carefully reviewed the clinical information of the patients included in the study, and their ISUP grading results were obtained from pathological diagnosis reports. We defined ISUP grades 1 and 2 as low-grade and ISUP grades 3 and 4 as high-grade for subsequent analyses^14^.

### ROI segmentation and radiomic feature extraction

We manually segmented the lesions of clear cell carcinoma and delineated the regions of interest (ROI) using ITK-SNAP (version 3.8.0). The ROI were defined during the corticomedullary phase (CMP), and this work was carried out by two radiologists with seven years of experience. A third radiologist with 12 years of experience reviewed their work and corrected any errors. The DICOM format data was then saved as NIfTI (.nii) format. We employed Pydiomics (version 3.1.0) in Python to extract radiomic features from the aforementioned data; this package is available on GitHub^15^. The extracted radiomic features included First Order Features, Shape Features (2D), Shape Features (3D), Gray Level Co-occurrence Matrix (GLCM) Features, Gray Level Size Zone Matrix (GLSZM) Features, Gray Level Run Length Matrix (GLRLM) Features, and Gray Level Dependence Matrix (GLDM) Features. The extracted features were saved in .csv format.

### Feature selection

We employed the Least Absolute Shrinkage and Selection Operator (LASSO) regression to perform dimensionality reduction on the extracted radiomic features, selecting important features for model construction and data analysis.

### Model development

The participants included in the study were randomly divided into training and testing sets in a 7:3 ratio. TCGA cohort were employed for the external validation set. The features selected by LASSO were standardized using the StandardScaler function. Four models—Logistic Regression (LR), Multilayer Perceptron (MLP), Support Vector Machine (SVM), and eXtreme Gradient Boosting (XGBoost)—were trained to predict the ISUP grading of ccRCC patients based on their CTU image CMP. The receiver operating characteristic (ROC) curve was plotted to assess model performance. Additionally, SHapley Additive exPlanations (SHAP) were employed to explain the importance of the classification features, and an interactive force plot was created to illustrate the model's decision-making process.

### Statistical analysis

The statistical analysis of this study was performed using Python (version 3.1.0), and all statistical tests were two-tailed, with p < 0.05 considered statistically significant.

## Results

### Patient characteristics

The baseline information of the study population is shown in **Table 1**.

### Radiomic feature extraction

A total of 1,218 radiomic features were extracted using the Pyradiomics package. We organized the names of the extracted radiomic features into a table, and detailed names of the radiomic features can be found in **Table S1**.

### LASSO regression

**Figure 3:**
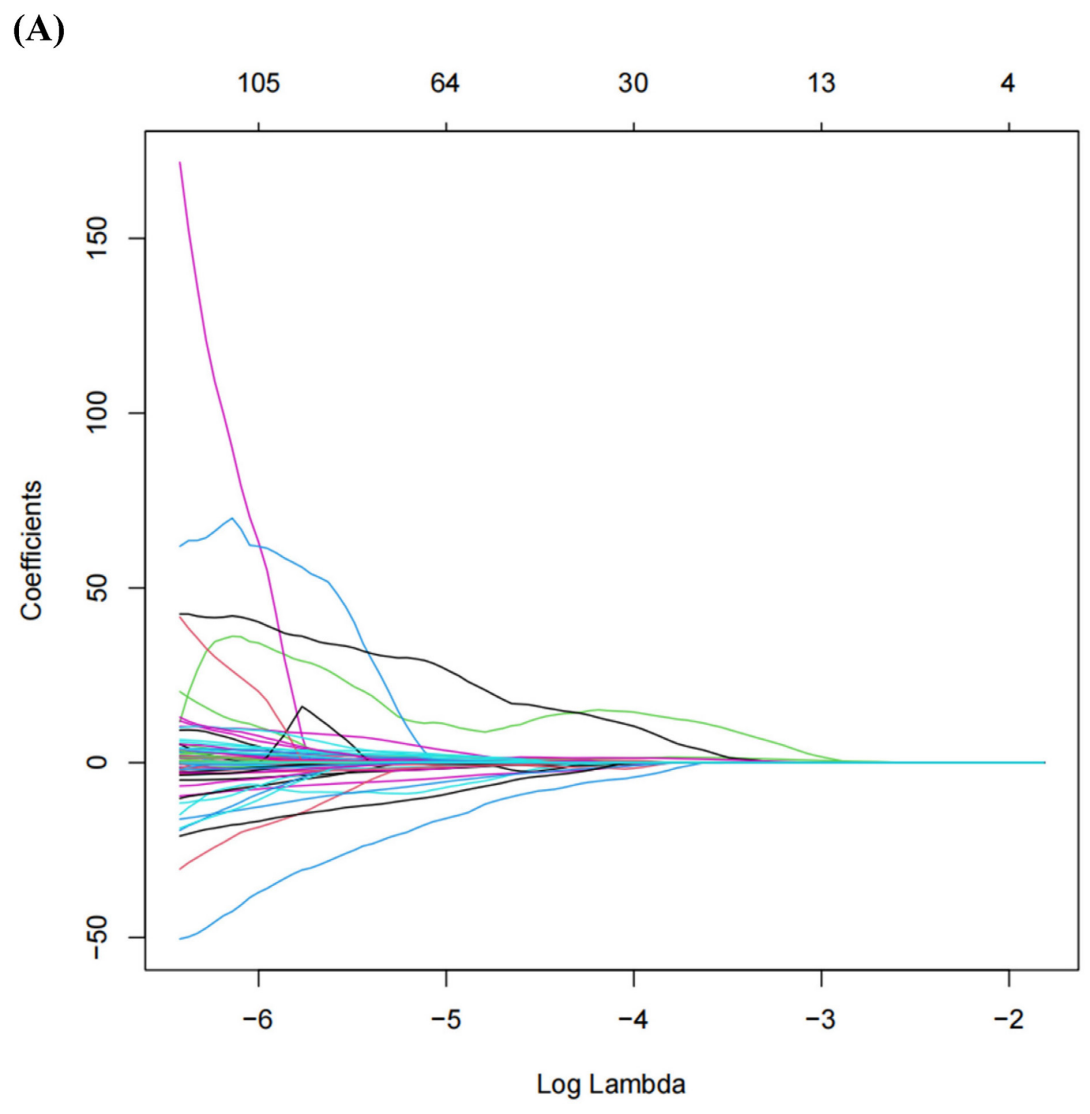

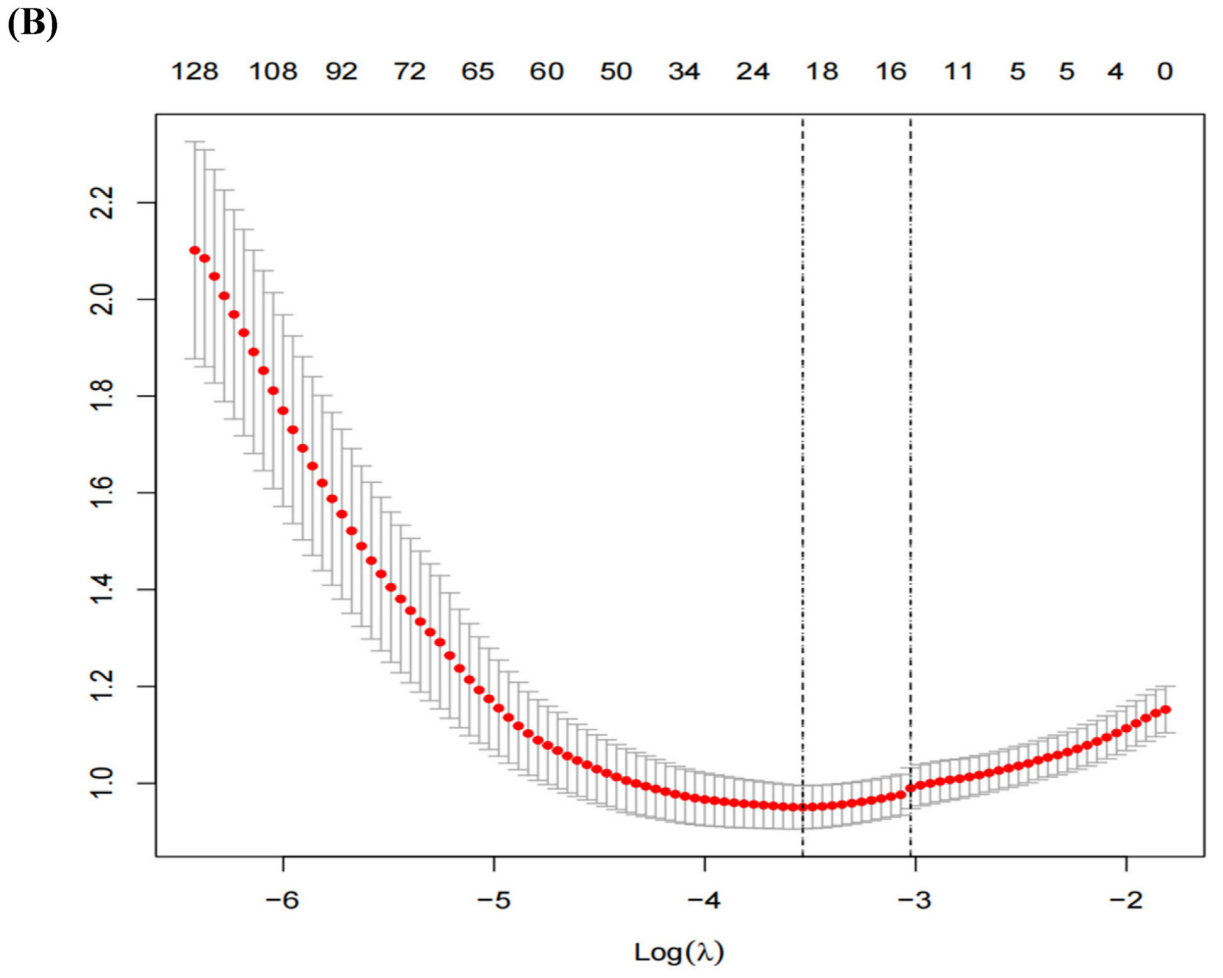
** (A)** LASSO Regression Coefficient Path Plot.** (B)** LASSO Regression Cross-Validation Curve.

A total of 1,218 radiomic features were filtered using LASSO regression, resulting in 20 features selected for model training (**Figure 3**). The radiomic features related to the ISUP grading of ccRCC identified by LASSO regression can be found in **Table 2**.

### The performance of machine learning models

**Figure 4:**
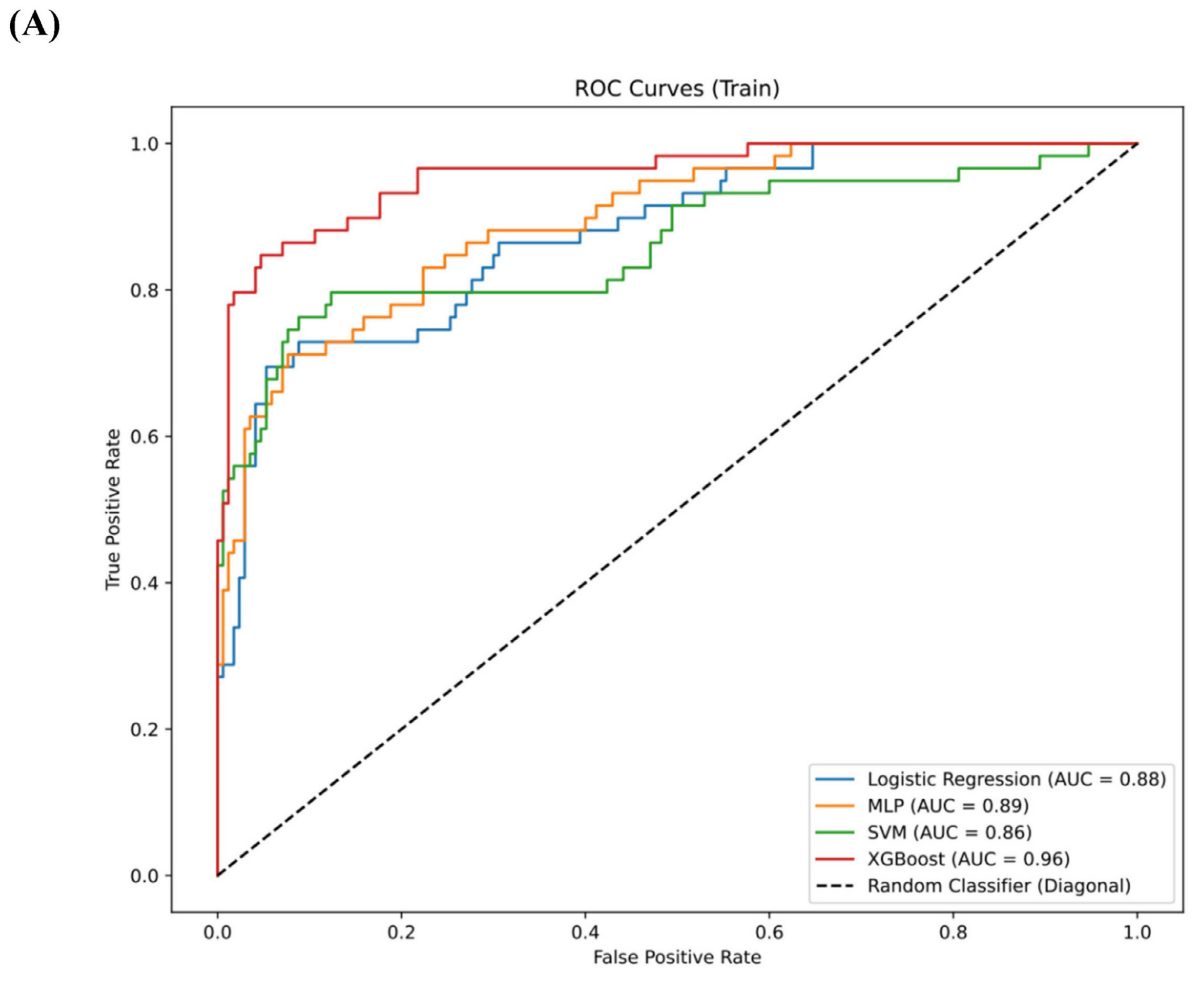

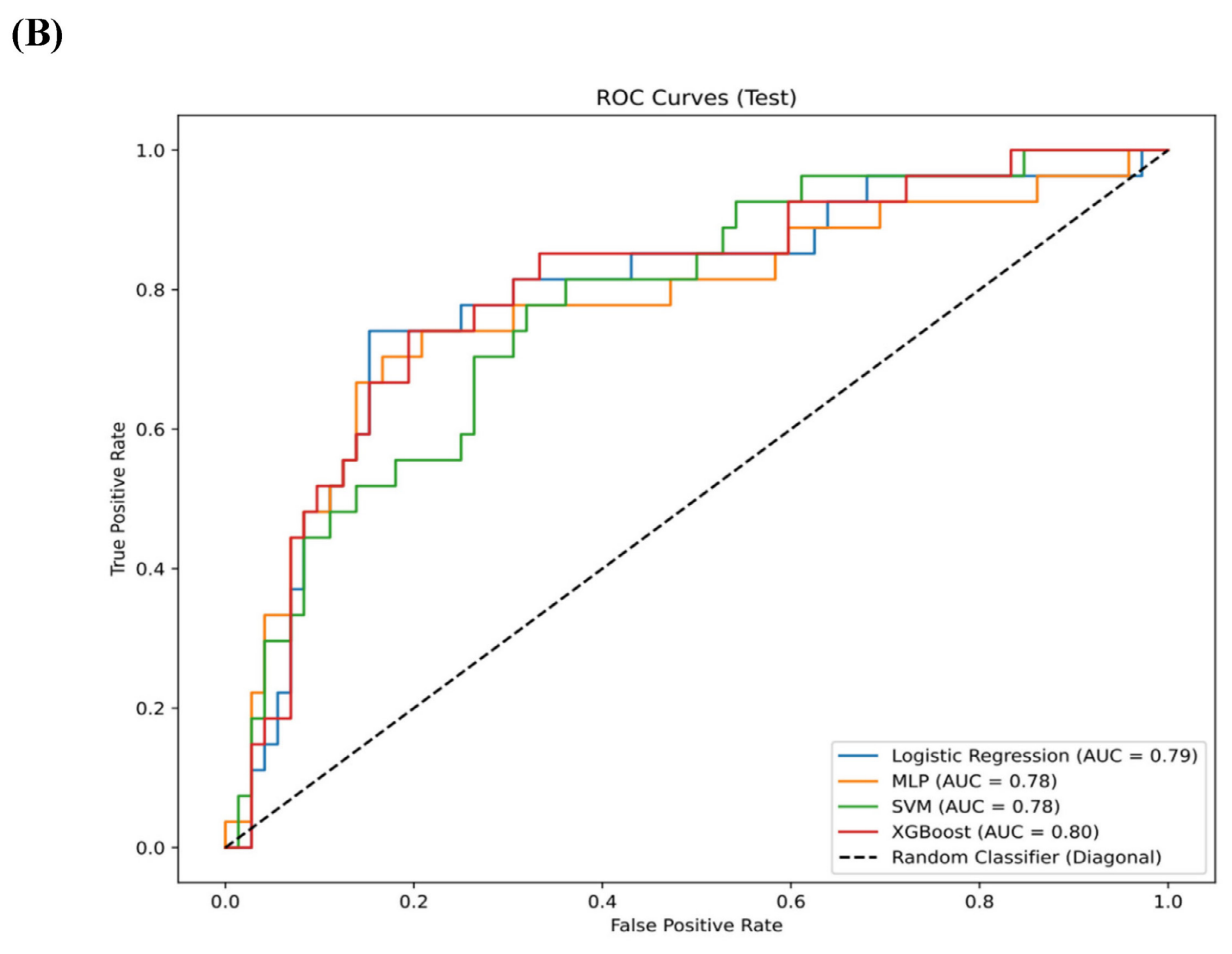

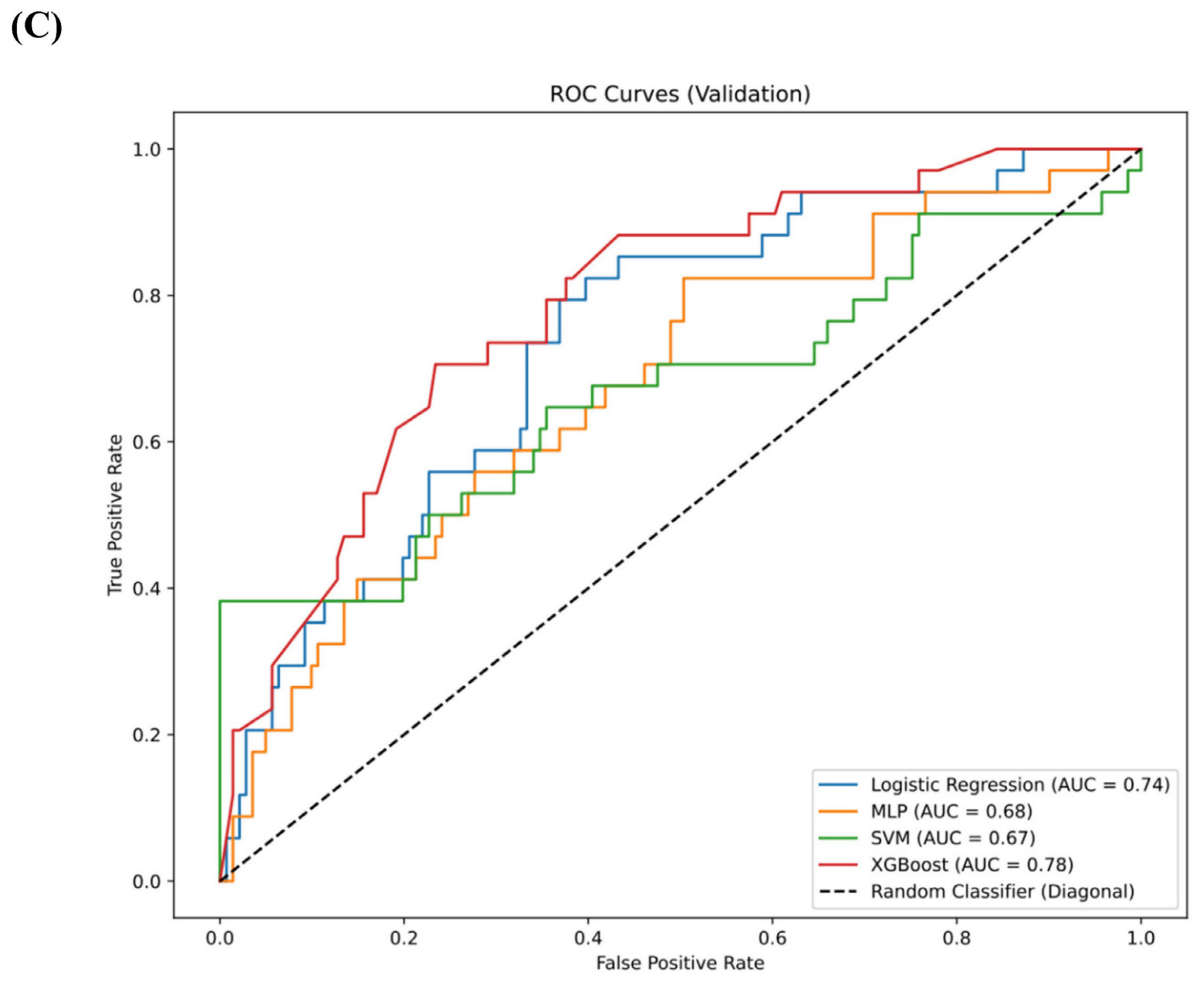
** (A)** The ROC curves for the four models—LR, MLP, SVM, and XGBoost—in the training set.** (B)** The ROC curves for the four models—LR, MLP, SVM, and XGBoost—in the testing set. **(C)** The ROC curves for the four models—LR, MLP, SVM, and XGBoost—in the validation set.

The comparison of clinical parameters between the training set and the testing set is shown in **Table 3**, and there is no significant statistical difference between them. (p>0.05) In the training set, AUC for the LR model was 0.88 (95% confidence interval [CI] 0.84, 0.91), the AUC for the MLP was 0.89 (95% CI 0.86, 0.93), the AUC for the SVM was 0.86 (95% CI 0.83, 0.90), and the AUC for XGBoost was 0.96 (95% CI 0.92, 0.99) (**Figure 4A**). In the testing set, the AUC for LR was 0.79 (95% CI 0.73, 0.85), the AUC for MLP was 0.78 (95% CI 0.72, 0.83), the AUC for SVM was 0.78 (95% CI 0.73, 0.82), and the AUC for XGBoost was 0.80 (95% CI 0.75, 0.85) (**Figure 4B**). In the validation set, the AUC for LR was 0.74 (95% CI 0.68-0.79), for MLP it was 0.68 (95% CI 0.63-0.73), for SVM it was 0.67 (95% CI 0.64-0.71), and for XGBoost it was 0.78 (95% CI 0.74-0.83) (**Figure 4C**). XGBoost demonstrated superior performance, achieving a sensitivity of 0.99 (95% CI 0.96, 1.00) in the training set, 0.92 (95% CI 0.88-0.97) in the testing set and 0.91 (95% CI 0.86,0.95) in validation set. The performance of the four models in both the training and testing sets is summarized in **Table 4**. We employed the Delong test to statistically evaluate the AUC values obtained from the ROC curves of various models. Within the training dataset, it was observed that the XGBoost model demonstrated superior performance compared to the LR, SVM, and MLP models (p<0.001).

**Figure 5:**
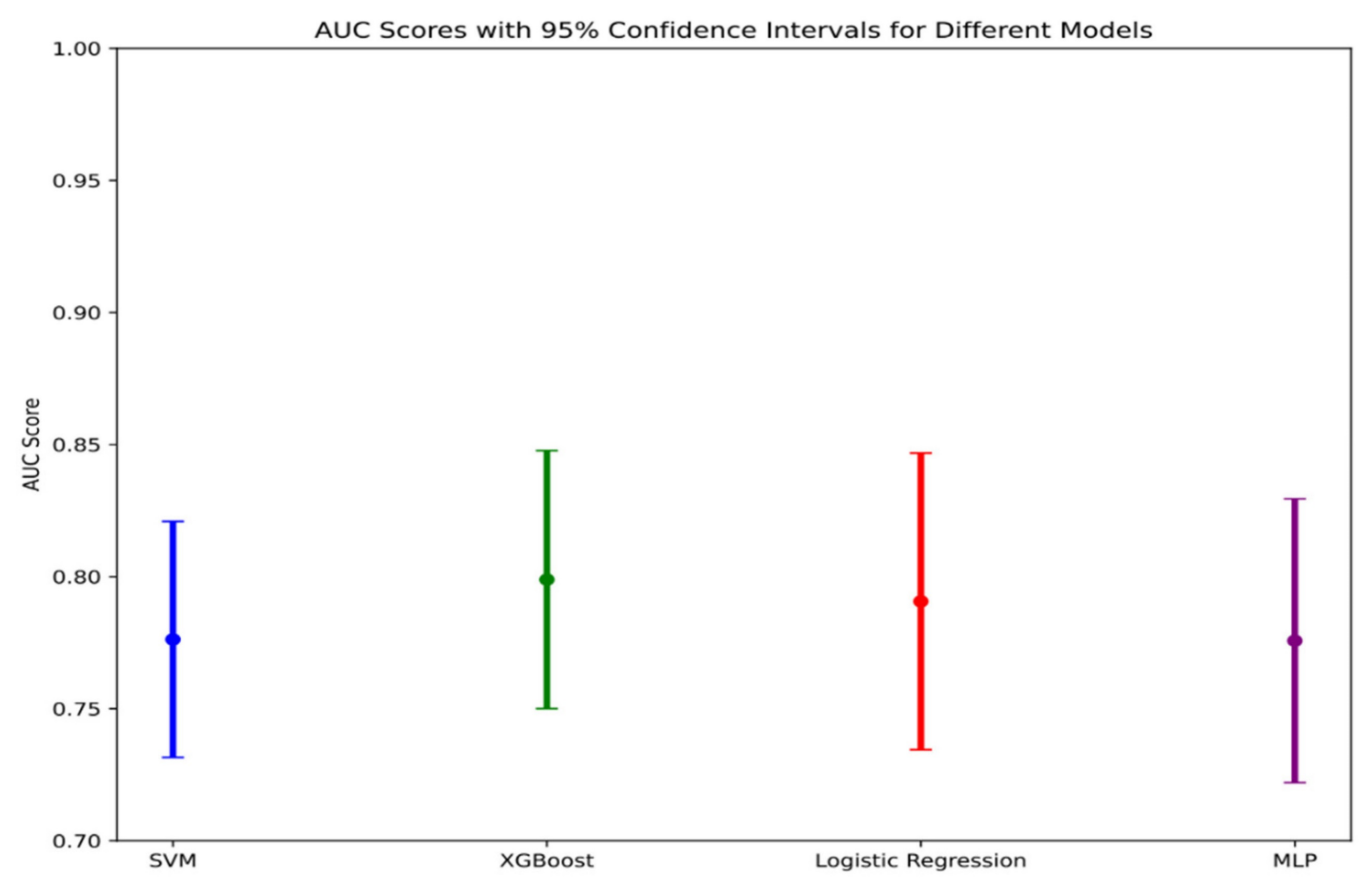
AUC Scores with 95% Confidence intervals for different models.

We plotted a data-class forest plot for the testing set, which visually demonstrated that XGBoost exhibited strong performance, with a narrower confidence interval compared to the other models, indicating that this model performed stably on the testing set data (**Figure 5**).

### SHAP analysis

**Figure 6:**
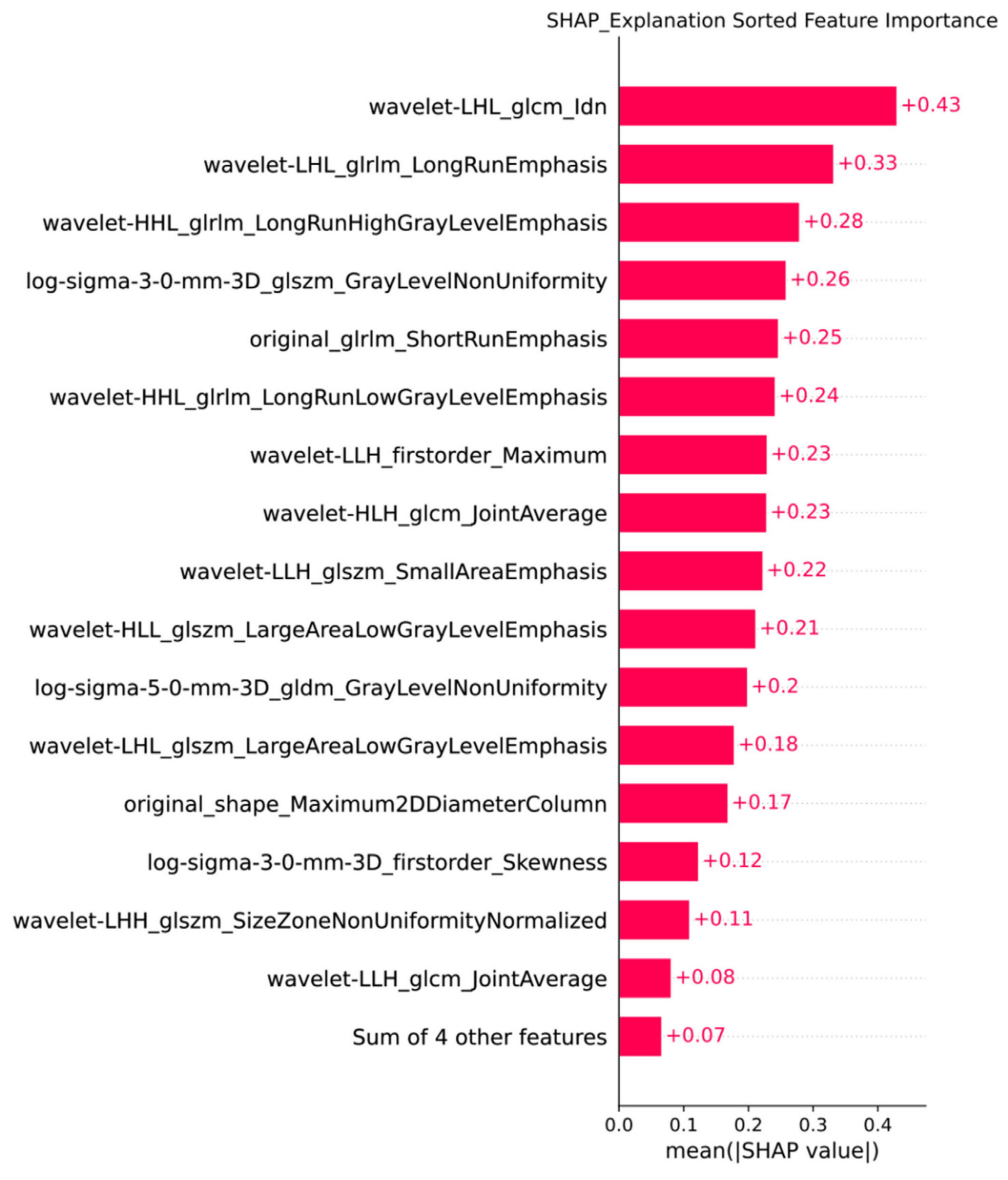
Feature Importance Bar Chart Generated by SHAP.

We utilized the `shap_values_Explanation` function to generate a feature importance bar chart for the 20 radiomic features of the trained XGBoost model, combining the least important features for a clearer representation of each feature's importance ranking (**Figure 6**).

## Discussion

The study pioneers a non-invasive approach to predict the ISUP grade of ccRCC by harnessing the power of artificial intelligence, with a particular emphasis on the XGBoost algorithm. It integrates radiomics feature extraction and advanced machine learning techniques to analyze CTU images. From these images, a comprehensive set of 1,218 features was extracted, and through the precision of LASSO regression, 20 key features were identified for model training. The XGBoost model's performance was rigorously evaluated across training, testing, and external validation sets, showcasing remarkable predictive capabilities, particularly with an impressive sensitivity of 0.99 in the training set. Furthermore, SHAP analysis illuminated the critical contributions of specific radiomics features to the classification process, opening new avenues for the exploration of potential biomarkers in ccRCC. This innovative method offers not only a valuable aid in clinical decision-making but also establishes a solid groundwork for the advancement of image-based cancer grading and the development of tailored treatment strategies in the future.

The WHO/ISUP grading system has been widely adopted for the pathological analysis of ccRCC. Its repeatability and strong correlation with clinical outcomes make it preferable to the Fuhrman grading system^16^, ^17^.

Research has shown that the WHO/ISUP grading system correlates with outcomes and tumor biological behavior in ccRCC patients^18^. Since treatment strategies differ between high-grade and low-grade renal cancers, early differentiation and diagnosis of ccRCC can significantly benefit treatment strategy selection and patient prognosis^19^. Currently, preoperative diagnosis of ccRCC mainly relies on percutaneous renal biopsy; however, this method can introduce trauma and may not accurately identify the pathological grading of ccRCC^20^-^22^.

As a result, numerous researchers have undertaken preliminary investigations into predicting the ISUP grading of clear ccRCC using medical images. For instance, Li, Q. *et al.* employed a radiomic model based on multiparametric MRI to predict the ISUP grading in ccRCC patients^14^. Similarly, Chen, R. *et al.* demonstrated favorable outcomes using a multi-sequence MRI-based radiomic model for preoperative prediction of ISUP grading in ccRCC^23^. Additionally, Chen, Y.F. *et al.* found that ultrasound-based radiomics can effectively predict the ISUP grading of ccRCC^24^.

In this study, we aimed to use CTU-based radiomic technology to predict the ISUP grading of ccRCC. The machine learning models we trained showed strong performance on both the training and testing sets, with the XGBoost model demonstrating particularly stable results across both datasets. The region of interest (ROI) delineation was conducted during the CMP phase, effectively minimizing the introduction of confounding biases. Using the LASSO model, we selected 20 radiomic features for training, leveraging L1 regularization^25^. By standardizing the data prior to model training, we enhanced both the stability and accuracy of our model's performance.

Machine learning models are often referred to as "black boxes," and interpretability has long been a challenge. We addressed this issue through SHAP, which enabled us to rank the importance of the radiomic features contributing to the model.

Although the four models we trained, particularly the XGBoost model, demonstrated strong performance in both the training and testing sets, our study has several limitations. First, it was conducted within a single cohort, and the sample size was insufficient, which may restrict the generalizability of our trained model. Second, we did not have a separate external cohort for validation, which poses challenges to the model's robustness and generalizability. We aim to conduct large-sample, multicenter, prospective clinical studies to enhance the generalizability of our model and provide a non-invasive and efficient solution for predicting the nuclear grading of ccRCC.

## Conclusion

This study reported a novel non-invasive method for predicting the ISUP nuclear grading system of ccRCC from CTU images, providing physicians with a new strategy to enhance clinical management and improve patient outcomes.

## Supplementary Material

Supplementary table.

## Figures and Tables

**Table 1:** Clinical and pathological characteristics of ccRCC patients included in the RHWU and TCGA cohort.

Characteristics	RHWU (*N* = 328)	TCGA (*N =* 175)
Age (years)	59 (25,84)	59 (26,88)
Gender		
Female	124 (37.8%)	64 (36.5%)
Male	204 (62.2%)	111 (63.5%)
pT stage		
pT1	224 (68.3%)	92 (52.5%)
pT2	40 (12.1%)	19 (10.9%)
pT3	51 (15.6%)	61 (34.9%)
pT4	13 (4.0%)	3 (1.7%)
pN stage		
pN0	326 (99.4%)	74 (42.3%)
pN1	2 (0.6%)	3 (1.7%)
pNx	0 (0%)	98 (56.0%)
pM stage		
pM0	326 (99.4%)	144 (82.3%)
pM1	2 (0.6%)	25 (14.3%)
pMx	0 (0%)	6 (3.4%)
pTNM stage		
Stage I	225 (68.6%)	89 (50.9%)
Stage II	39 (11.9%)	15 (8.6%)
Stage III	51 (15.5%)	44 (25.1%)
Stage IV	13 (4.0%)	27 (15.4%)
ISUP		
Low	242 (73.8%)	71 (40.1%)
High	86 (26.2%)	104 (59.9%)

**Table 2:** Radiomics features associated with ISUP grading selected by LASSO regression.

Signature	Coefficients
original_shape_Maximum2DDiameterColumn	0.00222852737199394
original_glcm_Idn	10.1546499325103
original_glrlm_ShortRunEmphasis	-0.132918290548955
original_glszm_GrayLevelNonUniformity	0.000411156981777663
log-sigma-3-0-mm-3D_firstorder_Skewness	-0.00178145972896154
log-sigma-3-0-mm-3D_glszm_GrayLevelNonUniformity	0.000879435041237894
log-sigma-5-0-mm-3D_gldm_GrayLevelNonUniformity	8.17007542499914e-07
wavelet-LLH_firstorder_Maximum	0.00113570868341896
wavelet-LLH_glcm_JointAverage	0.0060224563563358
wavelet-LLH_glszm_SmallAreaEmphasis	1.434207623494
wavelet-LHL_glcm_Idn	2.08239378405782
wavelet-LHL_glrlm_LongRunEmphasis	0.171694741441655
wavelet-LHL_glszm_LargeAreaLowGrayLevelEmphasis	4.61024679022084e-07
wavelet-LHH_glszm_SizeZoneNonUniformityNormalized	0.87375601873421
wavelet-HLL_glszm_LargeAreaLowGrayLevelEmphasis	6.48325519507287e-07
wavelet-HLH_glcm_JointAverage	0.0665272864500797
wavelet-HLH_glrlm_ShortRunHighGrayLevelEmphasis	0.0167593670054085
wavelet-HHL_glrlm_LongRunHighGrayLevelEmphasis	-0.000162138701876053
wavelet-HHL_glrlm_LongRunLowGrayLevelEmphasis	0.10348165095976
wavelet-LLL_gldm_LargeDependenceLowGrayLevelEmphasis	0.0486078683527515

**Table 3:** Comparison of clinical parameters between training set and testing set.

Variable	Overall, N = 328^1^	Training set, N = 230^1^	Testing set, N = 98^1^	p-value^2^
Gender				0.084
Female	124 (38%)	80 (35%)	44 (45%)	
Male	204 (62%)	150 (65%)	54 (55%)	
Age	59 (52, 66)	58 (51, 66)	62 (55, 66)	0.051
pTNM stage				0.081
I	225 (69%)	166 (72%)	59 (60%)	
II	39 (12%)	22 (9.6%)	17 (17%)	
III	51 (16%)	35 (15%)	16 (16%)	
IV	13 (4.0%)	7 (3.0%)	6 (6.1%)	
ISUP Grade				0.15
Low-grade	242 (74%)	175 (76%)	67 (68%)	
High-grade	86 (26%)	55 (24%)	31 (32%)	

^1^Median (IQR) or Frequency (%) ^2^Pearson's Chi-squared test; Wilcoxon rank sum test; Fisher's exact test

**Table 4:** Model performance summary.

Data Set	Model	AUC (95%CI)	Sensitivity (95%CI)	Accuracy (95%CI)
Training	LR	0.88 (0.84,0.91)	0.95 (0.92,0.98)	0.87 (0.81,0.92)
	MLP	0.89 (0.86,0.93)	0.95 (0.91,0.98)	0.86 (0.83,0.90)
	SVM	0.86 (0.83,0.90)	0.98 (0.94,1.00)	0.87 (0.82,0.91)
	XGBoost	0.96 (0.92,0.99)	0.99 (0.96,1.00)	0.93 (0.88,0.97)
Testing	LR	0.79 (0.73,0.85)	0.89 (0.83,0.94)	0.77 (0.72,0.81)
	MLP	0.78 (0.72,0.83)	0.89 (0.85,0.92)	0.79 (0.75,0.82)
	SVM	0.78 (0.73,0.82)	0.96 (0.93,0,98)	0.77 (0.73,0.81)
	XGBoost	0.80 (0.75,0.85)	0.92 (0.88,0.97)	0.80 (0.74,0.87)
Validation	LR	0.74 (0.68,0.79)	0.85 (0.81,0.88)	0.80 (0.75,0.84)
	MLP	0.68 (0.63,0.73)	0.73 (0.68,0.79)	0.78 (0.72,0.84)
	SVM	0.67 (0.64,0.71)	0.79 (0.76,0,83)	0.74 (0.69,0.78)
	XGBoost	0.78 (0.74,0.83)	0.91 (0.86,0.95)	0.86 (0.82,0.89)
